# Use of Carbon Fiber Implants to Improve the Safety and Efficacy of Radiation Therapy for Spine Tumor Patients

**DOI:** 10.3390/brainsci15020199

**Published:** 2025-02-14

**Authors:** Fred C. Lam, Santosh Guru, Deyaldeen AbuReesh, Yusuke S. Hori, Cynthia Chuang, Lianli Liu, Lei Wang, Xuejun Gu, Gregory A. Szalkowski, Ziyi Wang, Christopher Wohlers, Armine Tayag, Sara C. Emrich, Louisa Ustrzynski, Corinna C. Zygourakis, Atman Desai, Melanie Hayden Gephart, John Byun, Erqi Liu Pollom, Elham Rahimy, Scott Soltys, David J. Park, Steven D. Chang

**Affiliations:** 1 Department of Neurosurgery, Stanford University School of Medicine, Stanford, CA 94305, USA; fredlam@stanford.edu (F.C.L.); sg928@cam.ac.uk (S.G.); abureesh@stanford.edu (D.A.); yshori@stanford.edu (Y.S.H.); atayag@stanfordhealthcare.org (A.T.); saraemrich@stanfordhealthcare.org (S.C.E.); lustrzynski@stanfordhealthcare.org (L.U.); atman@stanford.edu (A.D.); mghayden@stanford.edu (M.H.G.); djpark@stanford.edu (D.J.P.); 2 Department of Radiation Oncology, Stanford University School of Medicine, Stanford, CA 94305, USA; chuangc@stanford.edu (C.C.); llliu@stanford.edu (L.L.); leiwang@stanford.edu (L.W.); xuejungu@stanford.edu (X.G.); gszalkow@stanford.edu (G.A.S.); zwang@stanfordhealthcare.org (Z.W.); cwohlers@stanfordhealthcare.org (C.W.); byunj@stanford.edu (J.B.); erqiliu@stanford.edu (E.L.P.); rahimy90@stanford.edu (E.R.); sgsoltys@stanford.edu (S.S.)

**Keywords:** spine tumors, spine oncology, spine metastasis, spine surgery, titanium pedicle screws, carbon fiber PEEK pedicle screws, stereotactic radiosurgery, cyberknife, gammaknife, proton beam radiation therapy, SBRT, neurosurgery, neuro-oncology

## Abstract

Current standard of care treatment for patients with spine tumors includes multidisciplinary approaches, including the following: (1) surgical tumor debulking, epidural spinal cord decompression, and spine stabilization techniques; (2) systemic chemo/targeted therapies; (3) radiation therapy; and (4) surveillance imaging for local disease control and recurrence. Titanium pedicle screw and rod fixation have become commonplace in the spine surgeon’s armamentarium for the stabilization of the spine following tumor resection and separation surgery. However, the high degree of imaging artifacts seen with titanium implants on postoperative CT and MRI scans can significantly hinder the accurate delineation of vertebral anatomy and adjacent neurovascular structures to allow for the safe and effective planning of downstream radiation therapies and detection of disease recurrence. Carbon fiber-reinforced polyetheretherketone (CFR-PEEK) spine implants have emerged as a promising alternative to titanium due to the lack of artifact signals on CT and MRI, allowing for more accurate and safe postoperative radiation planning. In this article, we review the tenants of the surgical and radiation management of spine tumors and discuss the safety, efficacy, and current limitations of CFR-PEEK spine implants in the multidisciplinary management of spine oncology patients.

## 1. Introduction

**Figure 1:**
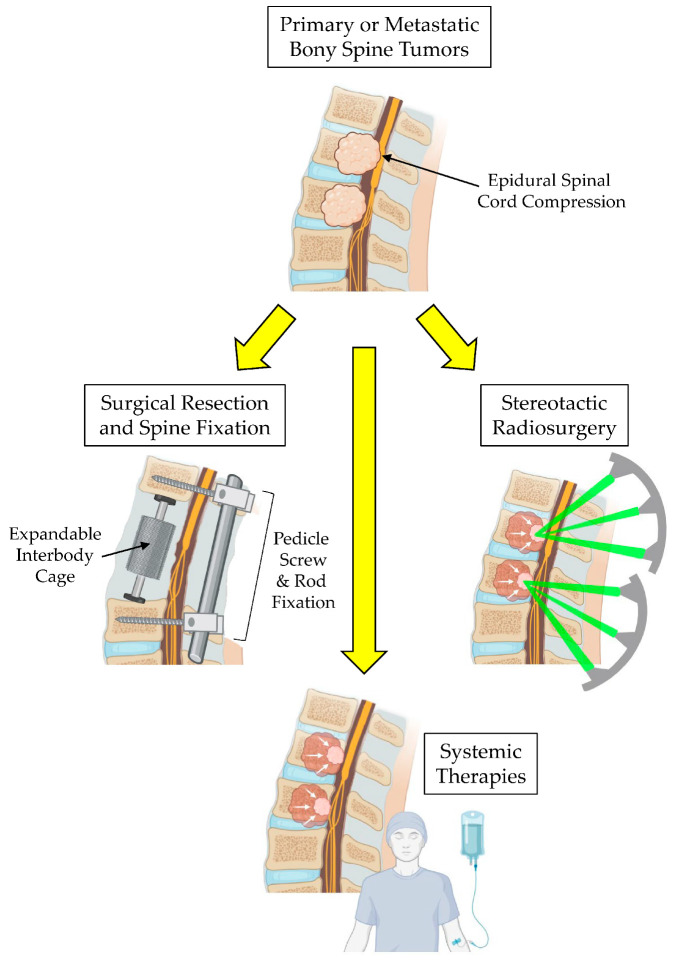
Multimodal therapy for the treatment of bony spine tumors.

Bony tumors of the spine, whether they be primary or secondary (metastatic) in nature, pose significant management challenges [1,2]. They create considerable morbidity for patients by causing pain and potential structural instability, which can lead to neurological deficits requiring urgent surgical decompression of the spinal cord and vertebral column fixation [3]. Primary bony spine tumors (PBSTs) are rare and account for approximately 5% of all primary bone tumors [4]. Eighty percent of PBSTs are benign and often do not require active clinical management [5]. Malignant PBSTs, including osteosarcoma, chondrosarcoma, Ewing’s sarcoma, chordoma, and plasmacytoma, comprise the remaining 20% of PBSTs [6]. These tumors are painful, present with radiculomyelopathy from nerve root and/or spinal cord compression, are locally aggressive, and often require surgical management, which can be associated with morbidity as high as 35% [7]. Metastatic bony spine tumors (MBSTs) are the most common vertebral column tumor in adults with incidences as high as 70–90% in lung, breast, prostate, and renal cell cancer patients [8]. As with PBSTs, patients presenting with intractable pain, progressive neurologic deficits, and/or spinal deformity require surgical management. In the current era of systemic targeting and chemotherapies combined with stereotactic radiotherapy, the management of malignant bony spine tumors requires a multidisciplinary team of surgical oncologists alongside medical and radiation oncologists to ensure the prompt and comprehensive treatment of these aggressive lesions (Figure 1) [6]. While we recognize that systemic chemotherapies and targeted therapies are important and integral components for treating patients with BSTs, for the purposes of this review article, we feel that this treatment subtopic is vast in and of itself and goes beyond the scope of this review. Therefore, we would like to focus this review article on discussions surrounding surgical treatment and radiation treatment paradigms for BSTs with a discussion on the emerging uses of carbon fiber-reinforced polyetheretherketone (CFR-PEEK) implants used by spine surgeons during tumor surgery as a means of enhancing the downstream radiation therapy planning of radiation oncologists to improve treatment outcomes.

## 2. Imaging Work-Up of Bony Spine Tumors

Radiographic imaging is key in the staging and management of spine tumors. Conventional X-ray radiographs are sensitive in detecting changes in bone mineral density caused by changes in calcium concentration in bone, which occurs during neoplastic bony invasion [9]. Anteroposterior and lateral weight-bearing X-ray radiographs provide rapid assessments of the spinal alignment under physiologic axial loads, especially when patients complain of persistent back pain and progressive radicular symptoms [10]. However, X-ray radiographs lack the resolution to identify and superimpose lesions within complex anatomy [11].

The hyper-density of calcium signals seen on CT is pivotal to delineate bony anatomy and assess the amount of bony destruction, spinal column destruction, and consequent spinal instability [12]. High-resolution multidetector CT scanners with 16 slices and above produce good cross-sectional displays and allow for multidimensional 2D and 3D reconstructions in complex anatomical locations [9]. CT angiography is also useful in identifying critical vascular structures surrounding and/or feeding these osseous lesions, such as the supra-aortic trunk or the artery of Adamkiewicz [13]. It is also instrumental in obtaining CT-guided bony biopsies. The ability to use CT to assess the integrity of the vertebral body, pedicles, and posterior spinal elements further allows for pre-surgical planning for spinal column reconstruction and stabilization after tumor resection [14]. However, the CT scan is not sensitive enough to detect the microperiosteal reactions of bony spine tumors [15,16], nor is it able to characterize non-mineralized matrix, soft tissue or medullary involvement, or the degree of epidural extension or canal compromise [9]. MRI is the preferred modality to overcome these limitations.

MRI is the most sensitive and specific imaging modality in the assessment of spine tumors [17]. T1-weighted image (T1-WI) sequences are ideal for delineating anatomy and for evaluating bone marrow invasion, which appears hypointense on T1-WI [18,19]. Post-contrast T1-WI sequences also provide differentiation between cortical bone, marrow, and surrounding tissues [20]. T2-WI detects pathologic changes in which cells and extracellular matrix have increased water content, which allows for the detection of extra-osseous tumors, peritumoral edema, and surrounding normal tissues [21]. Fat suppression sequences on T2-WI such as short T1 inversion recovery (STIR) are helpful in differentiating the extent of bone marrow edema [22]. Gadolinium-based T1-WI post-contrast imaging can distinguish benign from malignant lesions in approximately 80% of cases [23]. Malignant tumors usually show restricted diffusion of water molecules on diffusion-weighted image (DWI) sequences and can be used to track therapeutic response with subsequent decreases in signal intensity [23]. T1-weighted sequences with and without gadolinium contrast, alongside T2-weighted and short-tau inversion recovery (STIR) sequences, allow the clinician to clearly visualize the contents of the thecal sac, neuroforamen, nerve root plexi, posterior ligaments, and soft tissue elements [24,25]. MBSTs are typically hypointense on T1, hyperintense on T2 and STIR, and enhanced with contrast [26].

^18^F-fluorodeoxyglucose positron emission tomography (^18^F-FDG-PET)/CT is helpful in differentiating between a benign from malignant lesion when CT or MRI scans are inconclusive [1]. In clinical scenarios where musculoskeletal tumors are associated with areas of necrosis, there is increased risk of sampling error and the underestimation of tumor grade. ^18^F-FDG-PET/CT can be used to guide the biopsy to sample the part of the tumor that most likely contains the highest histologic grade of tumor cells [27]. Furthermore, 18F-FDG-PET/CT can be used to detect metastases outside what is detected within the views of CT and MRI and has potential for detecting local tumor recurrence. Taken together, the power of combining multiple imaging modalities can effectively assist clinicians in achieving an accurate list of differential diagnoses for patients presenting with bony spine tumors.

## 3. Surgical Management of Bony Spine Tumors

Surgical resection remains the preferred treatment of choice for PBSTs to obtain local disease control. The Enneking classification system of benign and malignant musculoskeletal tumors, initially established in the 1970s and adopted from protocols for the surgical management of long bone tumors, served as a guide early on for selecting the extent of surgical resection [28]. However, this classification system was not sufficient to address the complexities of spinal column tumors, leading to the establishment of the Weinstein–Boriani–Biagnini (WBB) system in 1997 [29].

The WBB classification system divides the vertebral body into 12 radially equal segments in the axial plane; superficial and deep intraosseous involvement; and intradural or extradural extraosseous involvement. The WBB classification system standardizes the terminology used to describe the extent of resection amongst spine surgeons across hospitals and institutions. Advances in neuro-anesthesia, the availability of intraoperative neuromonitoring, and refinement in en bloc surgical techniques have led to significant decreases in perioperative morbidity associated with these en bloc resections, leading to reductions in local recurrence rates and tumor-associated mortality [30,31]. Indications for the surgical resection of osseous spine tumors include cytoreduction and tumor control; spinal cord decompression; and the restoration of spinal stability. PBSTs are resected with the aim of cure, while MBSTs are mainly resected for palliative symptom management. Surgical planning for all spine tumors should be discussed in a multidisciplinary tumor board with goals of integrating adjuvant treatments following surgery [3,32,33].

En bloc resection entails the removal of the entire bony tumor without violating its capsule encased by a continuous margin of healthy tissue [33]. The WBB staging system further defines “radical” margins in lesions that are contiguous with the epidural space and has been clinically validated to accurately predict tumor margins in the majority of patients [30,34,35]. Total en bloc spondylectomy was first described by Tomita and colleagues involving an en bloc laminectomy and the stabilization of the posterior column using pedicle screw instrumentation followed by a total vertebrectomy and anterior column reconstruction for tumors contained solely within the vertebral body [36,37]. This technique has since been widely adapted by spine surgeons worldwide with promising results in achieving local tumor control in carefully selected patients [38,39,40,41]. Studies have shown that recurrence rates are lower in wide en bloc resections compared to intralesional or marginal en bloc resections, especially for aggressive primary tumors [30,42], with rates of disease-free survival following en bloc resection reported to be 92.6%, 63.2%, and 43.9% at 1, 5, and 10 years, respectively, in a cohort of patients with PBSTs [34]. Patients with MBSTs treated with en bloc resections tended to fare less favorably, with disease-free survival reported to be 61.8%, 37.5%, and 0% at 1, 5, and 10 years, respectively [34], and local recurrence rates have been reported to be as low as 11% in one series [43].

Risk factors for local disease control and recurrence for MBSTs depend largely on the primary tumor of origin and status of systemic disease control. Prior irradiation to the tumor bed increases risks of local recurrence in both PBSTs and MBSTs, which is thought to be due to radiation-related changes to the peritumoral tissues affecting the clear delineation of tumor margins [43]. The intraoperative dural tear and >50% occupancy of the spinal canal are also predictive of local recurrence in patients with MBSTs. Achieving an en bloc resection may be of benefit in patients with radioresistant metastases, namely arising from renal cell, hepatocellular, colon, thyroid, and non-small-cell lung carcinomas as well as melanomas [44]. The extent of tumor resection and spinal cord decompression becomes particularly relevant given that most patients with MBSTs receive stereotactic radiosurgery to the postoperative tumor bed. Radiation planning requires the clear visualization of the spinal cord and nerve roots on postoperative CT and MRI.

The surgical paradigm surrounding the treatment of MBSTs has advanced significantly since the landmark randomized, multi-institutional, non-blinded trial published by Patchell and colleagues in 2005 comparing radiotherapy alone (30 Gy in 10 fractions) to circumferential spinal cord decompression and vertebral column stabilization (if required) followed by radiotherapy within 14 days after surgery [45]. Significantly more patients (84%) were able to walk after surgical decompression than in the radiotherapy group (57%) and maintained the ability to walk significantly longer than those in the radiotherapy group (median: 122 days vs. 13 days). The need for steroids and opioids was also significantly reduced in the surgical patients. Prior to Patchell’s study, surgeons were performing simple posterior lumbar decompressions or using external beam radiation therapy (EBRT) to treat MBST patients with epidural spinal cord compression (ESCC). A randomized prospective comparative study by Young and colleagues in the late 1990s showed that EBRT was nearly as effective as simple decompressive surgery in controlling pain, restoring motor and sphincter function [46]. However, many patients developed kyphotic deformities over time following their laminectomies, leading to significant morbidity. In contrast, the goal of surgery in the Patchell study was to provide immediate direct circumferential decompression of the spinal cord with the surgeons’ choice as to the approaches taken to achieve this decompression, with the stabilization of the spine following tumor resection if evidence of spinal instability was present. Techniques to restore stability included use of methyl methacrylate cement, body grafting, metallic screw, and rod stabilization. The removal of the MBST had a direct effect on restoring the patients’ ability to walk, whereas EBRT alone was insufficient to control tumor regrowth and continued ESCC. More importantly, surgery did not lead to an increased length of hospital stay, and 30-day mortality rates did not significantly differ between groups, but 30-day morbidity was significantly increased in the radiation group [45], leading to a paradigm shift toward surgery for the treatment of MBSTs.

In 2010, the Spine Oncology Study Group established the spinal instability neoplasia score (SINS) to determine which MBST patients would benefit from spinal stabilization surgery [47]. Bilsky and Smith subsequently established the neurological, oncological, mechanical, and systemic (NOMS) decision-making framework in 2006 for spine oncology surgeons [48]. The NOMS system incorporates several factors, including (1) neurologic (presence or absence of ESCC); (2) oncologic (radiosensitive vs. radioresistant primary); (3) mechanical (stable vs. unstable spine); and (4) systemic factors (good vs. poor surgical candidate). With the SINS and NOMS scoring systems, patients with MBSTs who present with high-grade ESCC and spinal instability should be considered for surgery [49]. The success at achieving local tumor control using these criteria in combination with postoperative SRS led to the concept of “separation surgery” in patients with high-grade MBSTs presenting with ESCC [50].

Advances in intraoperative spine navigation techniques to confirm pedicle screw placement have significantly decreased perioperative morbidity and surgical efficiency compared to previous free-hand technique guided by anatomical landmarks and intraoperative fluoroscopy [51,52,53,54]. Minimally invasive techniques, including percutaneous pedicle screw fixation and tubular and expandable retractor systems, allow for greater muscle sparing, decreasing perioperative pain, time for tissue healing, and rapid pain relief from pathologic fracture stabilization [55]. Vertebral column reconstruction can be achieved using strut grafts, and titanium or PEEK expandable cages, which allows for the correction of sagittal plane deformity and encourages osseous integration using biologics that can be inserted into the barrel of the expandable cage (Figure 1) [56,57].

Vertebroplasty and kyphoplasty procedures can provide anterior column support in patients with MBSTs that have not breached the posterior cortex of the vertebral body without ESCC as a means of controlling bony pain and reducing the risk of kyphotic deformity [58]. These minimally invasive procedures serve as useful adjuncts for radiotherapy-induced pathologic fractures at the treated level and are sometimes performed pre-radiotherapy in patients with high SINS scores and mechanical pain [59,60].

## 4. Radiation Therapy for Bony Spine Tumors

Radiation therapy is often a critical component of treatment for spine tumors, whether definitive, palliative, or postoperative, to address gross/microscopic residuals. Treatments were historically performed as simple 3D fields with conventional external beam radiotherapy (cEBRT), often in standard palliative doses (8 Gy in 1 fraction, 20 Gy in 5 fractions, or 30 Gy in 10 fractions, like in the Patchell study). With these treatments, adjacent normal tissues often received similar doses as for tumors, and thus, regimens were limited by normal tissue toxicity. With improving imaging and technology, it has become possible to deliver increasingly precise and ablative doses (higher biologically effective dose or BED) compared to for tumors with a tight gradient to spare normal tissues like the often adjacent and radiation sensitive spinal cord. These ablative/near-ablative treatments are performed in 1–5 days, minimizing the chance of inter-fraction anatomic changes for these highly precise plans. Terminology for these ablative/near ablative regimens include the umbrella term stereotactic body radiotherapy (SBRT), or stereotactic ablative radiotherapy (SABR), and, specifically when referring to treatments of the brain/spine, stereotactic radiosurgery (SRS, nominally 1 fraction, or 1 ‘treatment’) and fractionated SRS (2–5 fractions).

When technically amenable for limited-extent metastatic spine tumors, SRS is often preferred over cEBRT [24,61] for dose escalation to optimize local control given morbidity progression, particularly for patients with questionable efficacy/tolerability of systemic therapy, limited metastatic disease/longer prognoses, radiation-resistant histologies (sarcomas, renal cell carcinomas, gastrointestinal tumors, non-small-cell lung cancer, and melanoma) [62], or in the context of prior radiation whereby a priority is to deliver sufficient dose to tumor and spare the previously radiated spinal cord. In cases not clinically or technically amenable to SRS (i.e., very limited prognosis, poorly delineated tumor, extensive multilevel disease), especially for radiosensitive tumors (lymphoma/myeloma, breast, prostate), cEBRT may be sufficient, with future SRS as salvage.

SRS achieves high levels of local control, independent of disease histology (greater than 85% with median follow-up of more than 1 year) compared to cEBRT (less than 4 months, median benefit) for radioresistant tumor types [63,64]. It also allows for a shorter treatment time, which is of convenience to the patients, which may allow for less time off systemic therapy and can have anti-tumor benefits (i.e., potential faster pain/tumor response) [65]. Furthermore, while systemic therapies alone are effective at achieving local primary tumor control, they are less effective at achieving metastatic osseous disease control [66]. This can be overcome by combining SRS, which can render synergistic and/or additive treatment effects and make more traditionally radioresistant tumor types more radioresponsive [67].

Yamada and colleagues at the Memorial Sloan Kettering Cancer Center (MSKCC) in New York published their single-institution landmark case series of 657 MST patients (a total of 811 lesions) who were primarily treated with SRS between the years of 2003 and 2015 [68]. Patients were followed with CT, MR, or PET imaging every 3 to 6 months until death. Target volumes were defined according to the international consensus guidelines. Tumor histology, gross tumor volume (GTV), planning target volume (PTV), an absolute dose that covered 95% of the PTV (PTV D95), and the percentage of PTV covered by 95% of the prescribed dose (PTV V95) were analyzed in relation to the local control and time to local disease progression. Twenty-eight lesions progressed with a mean time to failure of 26 months (range, 9.7–57 months). The mean prescribed dose was 24 Gy (range, 16–26 Gy) to the isodose line that optimized tumor coverage and then normalized to 100%. Both GTV D95 and PTV D95 were significantly associated with local failure but not tumor histology, suggesting that even for patients with radioresistant tumor types, the delivery of a higher dose of SRS was important to achieve long-term outcomes [68]. More importantly, this study stressed the importance of adequate dose delivery to the GTV and PTV (using an actual absolute dose rather than just the prescribed dose) to achieve durable tumor control with a 2% risk of local failure *regardless* of tumor histology. This is in stark contrast to the failure of cEBRT to achieve significant local control in radioresistant MBSTs [63,66,69,70]. In addition, a maximal spinal cord dose constraint of 1400 cGy resulted in a low rate (0.42%) of myelopathy. This and other subsequent studies underscore the importance of being able to clearly define GTV, PTV, and organs at risk (OAR) contours using CT, MRI, and PET to enable adequate actual dose delivery to achieve maximal tumor control with low morbidity [71,72].

SRS in the postoperative or definitive treatment of PBSTs is a less robust but interesting avenue and may be considered for certain histologies/clinical scenarios for unresectable or recurrent tumors [73,74,75,76,77]. Proton beam radiotherapy (PBRT) and carbon ion radiotherapy (CIRT) are other treatment modalities used to treat PBSTs today. PBRT delivers doses with a pronounced peak of dose deposition (Bragg peak), ideal for sparing adjacent normal tissues and OARs [78]. PBRT may be advantageous for the dose escalation of spinal chordomas and primary sarcomas compared to photon-based therapy [79,80,81], with a meta-analysis suggesting that the treatment of chordoma patients using PBRT leads to long-term overall survival compared to SRS [82]. CIRT uses charged particles to deliver energy with a high dose drop-off similar to PBRT and has an even higher relative biological effectiveness compared to proton- or photon-based treatments [3]. Five-year local control rates for patients with unresectable spinal chordomas and sarcomas have been reported to be as high as 80%, suggesting that patients with PBSTs or highly resistant MBSTs may benefit from particle-based radiation therapy in the immediate postoperative period or if their tumor is unresectable [83,84]. This is particularly poignant in cases of unresected or partially resected chordomas of the spine, as these have been traditionally classified as being resistant to cEBRT at doses less than 60 Gy [85]. PBRT has been particularly beneficial in being able to deliver these highly conformed doses to the tumor, sparing adjacent tissues [86,87,88]. A study on 44 skull base chordoma and chondrosarcoma patients who were treated with PBRT in combination with cEBRT reported 3-year local control rates of 83.1% for chordomas and 90% for chondrosarcomas [89]. A second series of 40 patients with unresected chordoma treated with combination PBRT/cEBRT reported 85.4% 5-year local control, 81.9% overall survival, 89.4% disease-specific survival, and 20.2% distant failure [90]. Four local failures occurred at a median tumor dose of 77.4 Gy radiobiological equivalent (GyRBE). A median follow-up of 18 months demonstrated significant volumetric reduction in the total target volume (TTV) and the soft tissue target volume (STTV) within the first 24 months following initial treatment and in the subsequent follow-up period. The median maximum percentage volumetric reduction in the TTV and STTV was 43.2% and 70.4%, respectively. A study by Kabolizadeh and colleagues stressed the importance of using the volumetric radiographic evaluation of TTV and STTV as an indicator of tumor response and further supported combined high-dose PBRT with cEBRT for patients with unresected spine and sacral chordomas [90].

In light of the importance of high-dose delivery to osseous spine tumors, the International Spine Radiosurgery Consortium put out contouring and planning guidelines for spine SRS planning [91,92] as well as consensus guidelines for postoperative target contouring [93]. These guidelines underscore the importance of not simply treating the GTV, but it is equally important to treat a clinical target volume (CTV) defined by a margin of surrounding normal bone where a microscopic tumor spread through contiguous marrow spaces [2]. For patients with osseous tumors located in the cervical and thoracic spine, it is imperative to be able to clearly define the spinal cord on pre- and postoperative imaging to avoid radiation-induced myelopathy, as this can cause permanent paralysis and can be fatal when it occurs in the high cervical cord [94,95,96]. However, if the contours around the spinal cord for radiation avoidance are too generous, this could lead to the under-dosing of the epidural space, which has been previously shown to be a common pattern of treatment failure [97]. These findings highlight the importance of and reliance on high-quality CT, PET, MRI, and CT myelogram for proper spinal cord contouring [98].

Planning CT with less than 2 mm thick axial cuts is the workhorse for the radiation planning of osseous tumors for delineating bony anatomy, superimposed on a planning MRI using 1–2 mm thick slices for the contouring of soft tissue borders and identifying OAR, such as the spinal cord [99,100,101,102]. Imaging information is used for the voxel-based dose calculation and optimization of beam intensities and beam orientation. Suboptimal image quality can lead to errors in defining GTV, PTV, and CTV and incorrect dose delivery, which could cause tumor recurrence if under-delivered, or radiation toxicity, the unnecessary inclusion of health tissues in the irradiation field, and injury to OARs [61]. Inaccuracies in quantifying Hounsfield Units (HU) of structures on CT can also result in errors in the electron density or stopping power estimate, ultimately affecting dosimetry calculations [100,103].

**Figure 2:**
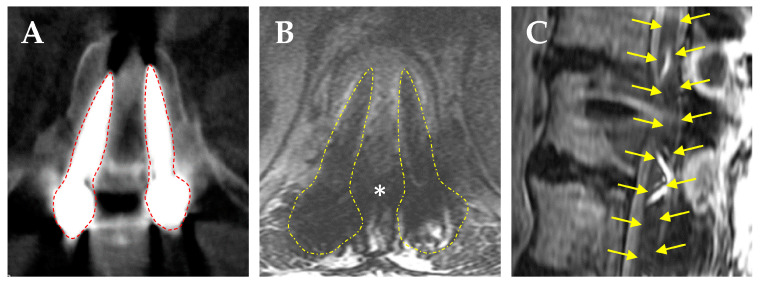
Radiographic artifact caused by titanium pedicle screws. (**A**) Axial CT scan shows large beam scatter caused by the titanium screws (red dashed lines). (**B**) Axial T2WI MRI showing scatter caused by the titanium screws (yellow dashed lines) obscuring the borders of the thecal sac (*). (**C**) Sagittal deformity of the thecal sac and spinal cord due to scatter caused by the titanium screws (yellow arrows outline a distorted image of the spinal cord).

With the increased use of metal implants for orthopedic hip and joint procedures, gold or amalgam tooth fillings, and metal alloy screws and rods for spine procedures, these high-density (Z) metals cause significant image artifacts in CT scans by causing beam hardening, scatter, and noise (Figure 2A) [104]. These artifacts cause inaccuracies in the calibration of CT HU to relative electron density for photon-based radiotherapy or to relative proton stopping power for proton radiotherapy [105]. The large difference in density and composition between these traditional high-Z metals compared to normal tissues can lead to high perturbation effects on radiation beams [106,107,108,109,110,111,112]. Dose calculations for PBRT are based on HU values assigned to the tissues within the treatment volume compared to a water-equivalent path length calibration curve; therefore, irradiation through metal implants should ideally be avoided to minimize inaccuracies in dosing that can adversely affect local disease control and normal tissue sparing [106,108,113,114]. The need to correct for these metal-related artifacts also increases planning time and may require multiple fields to improve dosimetric accuracy in patient with metallic spine implants undergoing PBRT [115,116], leading to potential delays in treatment [108,117]. Several techniques exist to reduce metal artifacts and improve dose calculation accuracy, including (1) megavoltage CT imaging [118]; (2) dual-energy CT scan [119,120,121]; (3) iterative reconstruction methods [122]; and (4) commercial orthopedic metal artifact reduction algorithms [123,124,125]. But they do not completing mitigate the artifactual effects of metal-based spinal implants.

Metal-induced artifacts are also seen on MRI, resulting in signal loss, pile-up artifacts, and geometric distortion (Figure 2B) [126,127]. This can significantly hinder the ability to contour CTV in postoperative patients, which according to SPIne response assessment in Neuro-Oncology (SPINO) group guidelines should include the pre-treatment bony and epidural tumor burden and surrounding bony structures at risk of microscopic tumor infiltration [93,128]. When the metal-induced artifact is too high to visualize the spinal cord (Figure 2B,C), CT myelography can be used to delineate the subarachnoid space; however, this entails another invasive procedure and risks of intrathecal contrast-allergic reactions [71,129]. This has led to the emerging use of low-density carbon fiber-reinforced polyetheretherketone (CFR-PEEK) pedicle screws and rods, which effectively decreases scatter artifacts and allows for more accurate postoperative radiation therapy planning [130].

## 5. The Use of Carbon Fiber-Reinforced Polyetheretherketone (CFR-PEEK) Pedicle Screws and Rods to Facilitate Post-Surgical Radiation Therapy of Bony Spine Tumors

CFR-PEEK spine implants are composed of composite carbon fiber sheets situated within a PEEK matrix compatible with osteoblastic integration [131,132]. They have an elastic modulus close to that of bone that decreases stress concentration at the bone–implant interface and confers implant stability [133,134]. CFR-PEEK pedicle screws have been shown to perform similarly to titanium (Ti) instrumentation regarding axial loading and compression [135] and have been found to have similar if not better stiffness, multicycle loading, and pull-out strength compared to Ti screws [136,137]. Newer iterations of CFR-PEEK pedicle screws and rods (i.e., CarboFix Spine Inc, USA; Icotec Medical Inc, Switzerland) utilize continuous carbon fiber strands reinforced with PEEK, which have been reported to have superior compressive force resistance and superior tensile strength compared to older generations of CFR-PEEK screws, and they also have comparable or superior bending load and stiffness compared to Ti screws [136].

**Figure 3:**
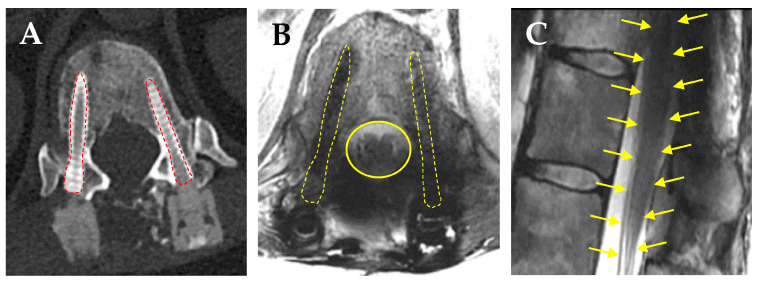
CFR-PEEK pedicle screws markedly reduces radiographic artifacts. (**A**) Axial CT scan shows minimal scatter caused by CFR-PEEK screws (red dashed lines). (**B**) Axial T2WI MRI showing clear borders of the thecal sac (yellow line), spinal cord, and nerve root anatomy. (**C**) Absence of sagittal distortion of the thecal sac, conus medullaris, and cauda equine (yellow arrows show a normal outline of the spinal cord, conus, and cauda equine).

A recent retrospective review by de Almeida and colleagues on consecutive patients who underwent thoracolumbar fusion for osseous spine metastases compared the pedicle screw artifact, spinal canal visualization, and sagittal distortion of the spinal cord on MRI among those who received Ti vs. CFR-PEEK pedicle screws [138]. They reported significant decreases in pedicle screw artifacts (5.8 mm CFR-PEEK vs. 13.2 mm Ti), much improved spinal canal visualization (19.2 mm CFR-PEEK vs. 15.5 mm Ti), and minimal sagittal distortion of the spinal cord at the screw level (0.5 mm CFR-PEEK vs. 1.9 mm Ti). A comparative cadaveric study by Kalasaukas and colleagues looked at CT and MRI artifacts using paired Ti pedicle screws, CFR-PEEK, and Ti screws, vs. paired CFR-PEEK screws and reported that imaging artifacts were clearly visible on CT but did not influence the visualization of intraspinal structures. Severe MRI artifacts prevented the evaluation of the spinal cord in 28% of paired Ti screws, 2% of CFR-PEEK and Ti screws, and 0% of paired CFR-PEEK screws [139]. Another retrospective cross-sectional comparison study on MRI artifacts caused by CFR-PEEK vs. Ti screws in patients with degenerative spine disease showed that a larger percentage of patients with CFR-PEEK screws had mean artifact-free postoperative MRI of their instrumented vertebral bodies (67.1%) compared to their Ti counterparts (48.3%) [140]. Furthermore, the visualization of the contents of the thecal sac in the lumbar spine was significantly improved in patients with CFR-PEEK screws. We have also observed a much lower amount of beam scatter on CT (Figure 3A) MRI (Figure 3B), and minimal sagittal cord distortion (Figure 3C) using CFR-PEEK screws. These studies support the superior capabilities of CFR-PEEK implants to decrease the amount of scatter on MRI and be able to better visualize OAR such as the spinal cord.

An ambispective cohort series by Boriani and colleagues in 2018 on 34 PBST and MBST patients subjected to thoracolumbar fixation using CFR-PEEK screws reported screw breakage in 1 of 232 implanted screws, 2 sacral screws loosening at 9 and 12 months, and 6 local recurrences found early due to implant radiolucency [132]. This preliminary study set the stage for establishing the safety profile of CFR-PEEK screws with regard to maintaining spinal stability and functional recovery. A retrospective review of 69 consecutive patients with osseous spine tumors treated using CFR-PEEK implants at MD Anderson Cancer Center who received adjuvant RT reported systemic disease progression in 28 patients, where 12 patients had local recurrence and the minimal imaging artifact caused by CFR-PEEK implants facilitated the postoperative RT planning and detection of local disease recurrence [141]. Ward and colleagues recently published a retrospective trial on 36 surgical MBST patients using CFR-PEEK implants who had postoperative SBRT planning using either an MRI or CT myelogram, reporting similar SBRT dosing and no significant survival between cohorts [142]. These studies all point toward the safety of using CFR-PEEK screws in achieving spinal stability after tumor resection and their ability to improve postoperative RT planning.

Several preclinical studies have shown benefits in using CFR-PEEK screws for PBRT planning. Nevelsky and colleagues measured the point dose differences between measured doses and Monte Carlo simulated doses for Ti vs. CFR-PEEK screw with an ultrathin tantalum coating using a 6 MV photon beam and found that the max dose perturbation was less than 5% for CFR-PEEK screws compared to greater than 30% for Ti screws [143]. Poel and colleagues compared Ti vs. CFR-PEEK screws using a phantom model to deliver proton beams to the spine and found that Ti constructs had up to 8% overdosing compared to ~5% with CFR/PEEK screws [144]. Shi and colleagues used phantom spine models instrumented with Ti, CFR-PEEK, or CFR-PEEK screws with a Ti tulip head and generated photon beam and proton beam treatment plans around a representative spinal chordoma target. They found that proton plans achieved 95% CTV and 90% OAR coverage with a tighter D_max_ for the spinal cord in spines instrumented with CFR-PEEK screws [145]. Similarly, Mastella and colleagues delivered proton and carbon ion Bragg Peaks to CFR-PEEK vs. Ti screws and measured transverse dose profiles using EBT3 films to evaluate beam perturbation and found that CFR-PEEK screws caused very slight beam perturbation compared to Ti screws with a lower degree of dose degradation during contouring [125]. Taken together, these studies show promise in the gradual integration of CFR-PEEK spine implants into the spine surgeon’s toolbox when there is a chance that their patients will require perioperative radiation therapy treatments to the spine.

Preclinical studies have consistently demonstrated that CFR-PEEK screws have significantly reduced imaging CT and MRI artifacts. These findings have translated well in clinical studies assessing the amount of imaging artifacts caused by CFR-PEEK compared to titanium screws, with studies favoring the use of CFR-PEEK screws when intending to increase the visualization of OARs, including the spinal cord. What is not so clear from the preclinical studies is the significance that CFR-PEEK screws have over titanium screws in creating more accurate dosimetry plans. The preclinical studies have shown decreased dose perturbation around CFR-PEEK implants with both photon and proton beam planning; however, the clinical studies have not convincingly demonstrated superiority in survival in patients with CFR-PEEK implants. As such, there is a need for higher-quality prospective clinical studies with longitudinal data documenting the superiority of CFR-PEEK implants in achieving local disease control to warrant widespread usage in spine oncology.

## 6. Recent Advances in Materials Used for Spinal Implants

Continued research on materials for spine surgery holds potential promise for new materials that may also have similar low-Z properties as CFR-PEEK with comparable advantages in reducing imaging artifacts while maintaining the structuring stability of the spine. The ideal biomaterial is one that is biologically inert/compatible, with a Young’s modulus similar to that of bone when implanted, with high tensile strength, stiffness, fatigue strength, and low artifacts on imaging [146]. While a full discussion on this topic would be beyond the scope of the review, we would like to highlight recent advances in biomaterials for spinal implants that yield promise for use in spine oncology to compliment downstream radiation oncology applications. While high-Z metals such as stainless steel, titanium, cobalt chromium, and nitinol are commonly used materials for spinal implants, as previously discussed, they cause significantly amounts of imaging artifacts. Newer materials, including ceramic, biodegradable materials such as polylactic acid (PLA), poly-lactide-co-glycolide (PLGA), and poly-_L_-lactide-co-d, _L_-lactide acid (PLLA), as well as three-dimensional (3D)-printed materials using polyetherketone ketone (PEKK) all have low-Z properties and less imaging artifacts, which may be favorable in helping downstream radiation oncology planning.

Materials such as ceramic doped with apatite-wollastonite (A/W) used in interbody cages have been shown to have similar biomechanical properties compared to PEEK [147] and could show promise in the future as this material could be incorporated into expandable cages for use in reconstructing the anterior column of the spine following oncologic vertebrectomy procedures. However, ceramic has the disadvantages of being brittle even after doping with A/W and has grafting issues, which may be overcome with coatings [146,148]. Similarly, biodegradable materials such as PLA, PLGA, and PLLA, when combined with hydroxyapatite to improve porosity for osteoconduction for interbody cages are biocompatible and have shown comparable biomechanical properties as PEEK cages; however, the clinical data for use in posterior lumbar interbody fusions have demonstrated failure rates as high as 26.4–50% [149,150,151]. As such, further research needs to be conducted to improve the durability of both ceramic and bioabsorbable spinal implants to garner traction for use in complex spine oncological surgeries.

Three-dimensional (3D) printing was used for the surgical planning of complex spine surgeries in the 1990s to create anatomical models for educational and surgical planning [152]. Purported advantages of using 3D printed constructs include improved patient outcomes, decreased radiation exposure for patients, and increased patient and resident education [153]. A recent biomechanical study comparing 3D printed PEKK cylindrical interbody implants to titanium and PEEK implants demonstrated comparable push-out strength compared to titanium-coated PEEK implants with “excellent” osteointegration and substantial bone growth at the 8- and 16-week time points. PEKK implants remained radiolucent and did not affect imaging quality, whereas titanium-coated implants were radiopaque and caused radiographic artifacts [154]. This preclinical study holds promise in seeing more widespread usage of 3D printed PEKK constructs for use in the spine oncology space. A recent presentation at the 2024 3rd annual Spine Tumor Symposium by oncologic spine surgeon Dr. Camilo Molina on his use of 3D printed spine constructs for use in reconstructing the oncological resection of spinal tumors nicely exemplifies this point [www.spinetherapysociety.org (accessed on 21 January 2025)]. For the ease of our readers, we summarize the use of various traditional and novel materials for spine oncology in Table S3.

## 7. Current Limitations and Future Outlook of Carbon Fiber-Reinforced Polyetheretherketone (CFR-PEEK) Pedicle Screws and Rods for Use in Spine Oncology

While CFR-PEEK screws are becoming increasingly adopted for used in spine oncology, there are several factors that currently limit their widespread use. A recent meta-analysis of clinical studies reported an overall implant-related complication rate of 7.8% in CFR-PEEK surgeries for patients with osseous spine metastases, which is slightly higher than previously reported studies using metallic implants [155,156,157]. Limited data exist on fusion rates using CFR-PEEK screws in the literature with only one retrospective study thus far comparing Ti implants to CFR-PEEK implants in MBST patients over a mean follow-up period of 14 months, which did not demonstrate any differences in outcomes or perioperative complications [158]. Longer-term follow-up studies with larger cohorts of patients using matched controls are needed to establish superiority over traditional Ti screws. In addition, the currently high costs and lead time associated with manufacturing CFR-PEEK screws currently limit the widespread use of these implants [157]. As the global multidisciplinary spine oncology community continues to embrace the use of CFR-PEEK implants, there will undoubtedly emerge more robust data to compare their efficacy in the treatment of patients with spine tumors.

## Data Availability

Not applicable.

## Supplementary Materials

The following supporting information can be downloaded at https://www.mdpi.com/article/10.3390/brainsci15020199/s1: Table S1: Preclinical studies characterizing carbon fiber screws; Table S2: Clinical studies characterizing carbon fiber screws; Table S3: Advances in materials used for spinal implants.
