# Supplementary material for: Use of Carbon Fiber Implants to Improve the Safety and Efficacy of Radiation Therapy for Spine Tumor Patients
Source: Brain Sci. 2025 Feb 14;15(2):199. doi: 10.3390/brainsci15020199 (PMC11852773; doi:10.3390/brainsci15020199)
Supplement: Supplementary file 1 [file brainsci-15-00199-s001.zip › Table S1 - Preclinical Studies.pdf]

Study	Design	Outcomes
**Ho et al, 2024**	Dose planning accuracy and postop CT image quality in CF-PEEK pedicle fixation systems compared to Ti.<br><br>Dose profiles through the screw, tulip, longitudinal axis of the screw acquired with radiochromic films and compared to a collapsed cone algorithm simulation, to measure dose agreement.<br><br>Image quality of postop CTs comparing 4 regions of interest around vertebrae and screws in water phantom models and previous planning CTs and comparing calculated artefact indexes.	CF-PEEK screws have non-inferior dosimetric prediction accuracy up to 50 mm beneath screw for collapsed-cone algorithm planning system.<br><br>Statistically significant reduction in absolute difference between calculated and measured dose at a depth of 2 mm beneath the screw.<br><br>Minimal attenuation with CF-PEEK relative to surrounding dose, extending to 50 mm beneath the screw.<br><br>Statistically significant improvement in CT imaging quality with reduced artifact index values in CF-PEEK vs Ti fixation.
**Nevelsky et al, 2017**	Measured point dose differences between measured doses and Monte Carlo simulated doses for Ti vs CF-PEEK vs CF-PEEK with ultrathin Ti coated screws using 6 MV photon beam using a solid water phantom.	Max dose perturbation < 5% for CF-PEEK vs > 30% for Ti screws. Ultra Ti coating had negligible effect.<br><br>Dose attenuation < 0.5% at depths of 1-6 mm when beam were passed across CF-PEEK screw vs 7.2-12.5% using Ti screw at same depths.
**Poel et al, 2020**	Phantom model study comparing Ti vs CF-PEEK using proton beam therapy.	CF-PEEK implant had 90% reduction of artifacts on CT images, significantly reducing time for artifact correction vs Ti-only implant.<br><br>Up to 8% overdose with Ti construct vs ~ 5% with CF-PEEK.
**Shi et al, 2022**	Spinal phantom models with a) No instrumentation; b) Ti; c) CF-PEEK; and d) hybrid CF-PEEK with Ti tulip head.<br><br>Measured CT iterative Metal Artifact Reduction.<br><br>Representative spinal chordoma target and associated OARs were contoured with 50 Gy prescription dose to the initial target volume, followed by 24 Gy boost using multi-field optimization proton plans and photon VMAT plans were made.	Proton plans achieved similar normal target coverage and OAR sparing. For initial CTV, ensured 95% coverage and 90% OAR coverage for normal and CF-PEEK plans. Uncertainty analysis of spinal cord Dmax showed tighter distribution for normal and CF-PEEK plans.<br><br>No difference in overall plan quality for photon vs proton when compared to normal spine regardless of type of implant.<br><br>For proton plan, larger difference for normal spine insert vs Ti insert.<br><br>For each insert scenario comparison between photon and proton plans, there was larger differences for OARs: heart and spinal cord.
**Kalasauskas et al, 2022**	Cadaveric imaging study of Ti-Ti vs C-Ti vs C-C pedicle screws using CT, 1.5T, and 3T MRI.<br><br>Axial T1- and T2-weighted sequences of T and L regions assessed for artifacts.<br><br>Artifacts classified as: 1) Not relevant; 2) Considerable; and 3) Severe.	92 screws and 178 artifact assessments made.<br><br>Artifacts clearly visible on CT but did not influence visualization of intraspinal structures.<br><br>Severe MRI artifacts hampering evaluation of the spinal cord found in 28% of Ti-Ti, 2% of C-Ti, and 0% of C-C screws.<br><br>Considerable MRI artifacts found in 47% of Ti-Ti, 10% of C-Ti, and 0% of C-C screws.

Abbreviations: CF/PEEK = carbon fiber/polyetheretherketone; Ti = titanium; CT = computed tomography; MV = megavoltage; OAR = organs at risk; Gy = gray; VMAT = volumetric modulated arc therapy; C = carbon; T = tesla; MRI = magnetic resonance imaging; CTV = clinical target volume.
