# Supplementary material for: Use of Carbon Fiber Implants to Improve the Safety and Efficacy of Radiation Therapy for Spine Tumor Patients
Source: Brain Sci. 2025 Feb 14;15(2):199. doi: 10.3390/brainsci15020199 (PMC11852773; doi:10.3390/brainsci15020199)
Supplement: Supplementary file 1 [file brainsci-15-00199-s001.zip › Table S2 - Clinical Studies.pdf]

Study	Design	Outcomes
**Ward et al, 2024**	Retrospective trial of 36 consecutive patients undergoing spinal fusion using CF-PEEK instrumentation for spine metastases with postop SBRT from 01/01/2022 to 04/03/2023.<br><br>Two cohorts: MRI vs CT-myelogram for postop SBRT planning.	CT-myelogram (N=11), MRI spine (N=25) for SBRT planning.<br><br>Median follow-up 145.5 days (13-530 days). No significant differences between baseline demographic, surgical characteristics, or SBRT dosimetry between MRI and CT-myelogram patients.<br><br>No significant differences between cohorts for survival (P=0.402).
**Alvarez-Breckenridge et al, 2023**	Retrospective review of 69 consecutive oncology patients treated with CF-PEEK implants at MD Anderson Cancer Center.	491 CF-PEEK pedicle screws implanted.<br><br>Adjuvant RT in primary tumors (N=8) and metastatic tumors (N=29).<br><br>28 patients from combined primary and metastatic cohorts experienced systemic disease progression.<br><br>12 patients with local recurrences.<br><br>Largest series of CF-PEEK implants demonstrates safe and effective spinal stabilization for patient with both primary and metastatic tumors.<br><br>Enhanced post-op imaging led to minimal imaging artifacts which facilitated postop RT planning and ability to detect local recurrence.
**Khan et al, 2023**	Systematic review on implant complications and RT benefits of CF-PEEK spinal implants for primary and metastatic spine tumors using articles that described patients with CF-PEEK pedicle screw fixation and a minimum of 5 patients.<br><br>Case reports and phantom studies were excluded.<br><br>11 articles with 326 patients (237 patients with CF-PEEK-based implants vs 89 with titanium-based implants).	Mean follow-up period of 13.5 months, 67.1% of tumors were metastatic.<br><br>Implant-related complications:<br>1) CF-PEEK 7.8%<br>2) Titanium 4.7%<br><br>Rate of pedicle screw fracture:<br>1) CF-PEEK 1.7%<br>2) Titanium 2.4%<br><br>Rate of reoperation:<br>1) CF-PEEK 5.7% due to implant failure or junctional kyphosis<br>2) Titanium 4.8% due to implant failure or junctional kyphosis<br><br>Four articles suggested implant artifact was reduced in the CF-PEEK group.<br><br>Local recurrence:<br>1) CF-PEEK 14.4%<br>2) Titanium 10.7%
**de Almeida et al, 2023**	Measurement of diameter of signal drop pedicle screw artifact and diameter of spinal canal free from artifacts (canal visualization) in consecutive patients who had spinal instrumentation followed by spinal SRS from June 2019 – May 2022.<br><br>Measurement of sagittal distortion of spinal cord at the screw level.	50 patients, 356 screws, 183 levels evaluated.<br><br>Pedicle screw artifact<br>- Ti = 13.2 mm<br>- CF-PEEK = 5.8 mm<br><br>Canal visualization<br>- Ti = 15.5 mm<br>- CF-PEEK = 19.2 mm<br><br>Sagittal distortion<br>- Ti = 1.9 mm<br>- CF-PEEK = 0.5 mm
**Fleege et al, 2020**	Retrospective cross-sectional comparison study of MRI artifacts using CF-PEEK vs Ti screws in degenerative spine disease.	N = 27 patients with Ti screws<br>N = 20 patients with CF-PEEK screws<br><br>Post-op MRI at 4 wks:<br>Ti mean artifact-free VB area = 48.3 ± 5%

	Surface of artifact-free VB area calculated as percentage of total vertebral body.<br><br>Assessability of the spinal cord, neuroforamina, pedicles screws, surrounding bony and soft-tissue structures.	CF-PEEK mean artifact-free VB area = 67.1 ± 5.6%<br><br>Assessability of L spine significantly improved for CF-PEEK screws for all measurements.<br><br>CF-PEEK pedicle screws exhibit smaller artifact areas on VB surfaces and their surrounding tissues.
**Mastella et al, 2017**	1) Proton and carbon ions spread-out Bragg Peaks were delivered to CF-PEEK and Ti screws using water phantoms.<br><br>Transversal dose profiles were acquired with EBT3 films to evaluate beam perturbation, with effects on image quality and reconstruction artifacts.<br><br>CT scans of CF-PEEK and Ti implants acquired and HU mean values evaluated in three regions of interest.<br><br>Implants and artifacts contoured in sample CT scans, together with a target volume to simulate a spine tumor.<br><br>Dose calculation accuracy assessed by comparing optimized dose distributions with Monte Carlo simulations.<br><br>2) Retrospective analysis of treatment plans of 9 real plans (7 CF-PEEK, 2 Ti implants) to evaluate dosimetric impact potentially occurring if improper management of the spine implant was carried out.<br><br>All patients received carbon ion radiation therapy in 11-16 fractions with relative biological effectiveness (RBE) equivalent prescription doses up to 76.8 Gy (RBE).<br><br>Gamma passing rates (GPs) evaluated separately for 9 clinical target volumes (CTVs) and for 13 organs at risk (OARs) in the treatment fields (4 spinal cords, 5 cauda equines, 4 esophagus).	1) Phantom Study Results:<br><br>CF-PEEK screws caused very slight beam perturbation compared to Ti screws: lower degree of dose degradation in case of contouring and/or set-up uncertainties.<br>CF-PEEK devices did not determine appreciable HU artifacts on CT images, improving image quality, final result, dose calculation accuracy.<br><br>2) Patient treatment plan results:<br><br>Mean GPs of all 7 CF/PEEK patient cases analyzed were 98.6 ± 1.3% for the CTVs, 100 ± 0.1% for OARs.<br><br>Mean GPs for 2 Ti patient cases were 89.0 ± 1.9% for the CTVs, 95.5 ± 5.5% for OARs.<br><br>Very good agreement in the presence of CF/PEEK implants (mean GPs > 98% for CTV and OARs) small dose deviations<br><br>Severe dose deviations found in CTVs in the presence of Ti implants.<br><br>Encourage use of CF-PEEK implants based on dosimetric investigation.
**Boriani et al, 2018**	Ambispective cohort series of 34 tumor patients (14 metastases, 20 primary, most recurrent) submitted to thoracic and lumbar spine fixation with CF-PEEK composite implants.<br><br>Oncology surgery was palliative decompression and fixation in 9 cases, tumor excision in 21, en bloc resection in 4.<br><br>Incidence of complications, changes in neurological status, local control, and survival.	1 out of 232 implanted screws broke.<br><br>2 sacral screws loosened at 9 and 12 months in multilevel constructs performed on multiple recurrent tumors.<br><br>6 local recurrences were found early due to implant radiolucency.<br><br>Clinical use of CF-PEEK screws are safe and comparable with Ti implants in terms of intraoperative complications, stability at weight bearing, and functional recovery.

Abbreviations: CF/PEEK = carbon fiber/polyetheretherketone; Ti = titanium; CT = computed tomography; MV = megavoltage; OAR = organs at risk; Gy = gray; VMAT = volumetric modulated arc therapy; C = carbon; T = tesla; MRI = magnetic resonance imaging; CTV = clinical target volume; SBRT = stereotactic body radiotherapy; RT = radiotherapy; HU = Hounsfield Unit; RBE = relative biological effectiveness; GP = gamma passing rates.
