# Supplementary material for: Use of Carbon Fiber Implants to Improve the Safety and Efficacy of Radiation Therapy for Spine Tumor Patients
Source: Brain Sci. 2025 Feb 14;15(2):199. doi: 10.3390/brainsci15020199 (PMC11852773; doi:10.3390/brainsci15020199)
Supplement: Supplementary file 1 [file brainsci-15-00199-s001.zip › Table S3 - Advances in Materials for Spinal Implants.pdf]

Material	Advantages	Disdavantages	Applications
Stainless Steel	Strong<br>Stiff<br>Inexpensive	Poor biocompatibility<br>High imaging artifacts	Use now mostly limited for use in rods for scoliosis correction
Titanium	Lightweight<br>Strong<br>Flexible<br>Biocompatible	Relatively expensive<br>Imaging artifacts	Screw<br>Rods<br>Cages<br>Plates
Polyetheretherketone	Lightweight<br>Flexible<br>Biocompatible<br>Low imaging artifacts	High Young's modulus<br>Some grafting issues but improved with coatings	Rods<br>Cages<br>Screws<br>Disc replacements
Cobalt Chromium	Strong<br>Flexible<br>Biocompatible	Relatively expensive<br>High imaging artifacts	Mainly used for pediatric scoliosis correction rods
Ceramic	Relatively inexpensive<br>Biocompatible<br>Water resistant	High Young's modulus<br>Low tensile strength<br>Poor crack resistance<br>Low fracture toughness<br>Brittle even with doping<br>Grafting issues but can be improved with coating/doping	Cages<br>Doped with apatite-wollastonite
Nitinol	Strong<br>Maintains shape memory	Relatively expensive<br>Stiffness may not be enough to maintain correction	Not widely used, may be used for pediatric scoliosis surgeries
Tantalum	High frictional characteristics	Very expensive<br>Stiffness may not be enough to maintain correction	Not frequently used due to price.<br>Largely been replaced by titanium.
Biodegradable Materials (ie. PLA, PLGA, PLDLLA, PLLA)	Biocompatible<br>Osteoconductive	Inferior biomechanical properties compared to permanent materials<br>Reported high failure rates of 26.4%-50% in posterior lumbar instrumented fusions	Interbody cages<br>ACDF plates and screws
3D Printed Materials (ie. PEKK)	Osteoconductive<br>Antibacterial properties	Few robust clinical studies	Interbody cages

Abbreviations: PLA = polylactic acid; PLGA = poly-lactide-co-glycolide; PLDLLA = poly-L-lactide-co-d, L-lactide acid; PLLA = poly-L-lactide acid); ACDF = anterior cervical discectomy and fusion; PEKK = polyetherketoneketone.
